# HIV-1 transmitted drug resistance in Shanghai, 2018–2024: population mobility shapes cross-population TDR transmission dynamics

**DOI:** 10.1080/22221751.2026.2731504

**Published:** 2026-09-18

**Authors:** Yuan Dong, Qianru Lin, Xuqin Wang, Changhe Liu, Xiaolei Yu, Wenqi Tang, Wanqing Feng, Xiaoning Lv, Yiqing Han, Qing Yue, Zhen Ning, Xin Shen, Xin Chen, Yanqiu Zhou, Min Chen, Huanyu Wu, Yi Lin

**Affiliations:** Shanghai Municipal Center for Disease Control and Prevention, Shanghai, People’s Republic of China

**Keywords:** HIV-1, transmitted drug resistance, molecular transmission network, phylogeography, population mobility

## Abstract

Transmitted drug resistance (TDR) poses a growing challenge to HIV control, particularly in highly mobile urban settings with complex transmission dynamics. We analyzed HIV-1 *pol* sequences from 7,538 newly diagnosed, treatment-naïve individuals in Shanghai (2018–2024), integrating genotypic resistance testing, molecular transmission network analysis, and time-scaled discrete phylogeographic inference. Individuals with Shanghai hukou were classified as Shanghai residents, whereas those without Shanghai hukou were categorized by residence as resident migrants (residing in Shanghai) or temporary migrants (residing outside Shanghai). Overall TDR prevalence was 5.1% (95% CI: 4.6%–5.6%), and was higher among individuals with recent infections (7.3%, 95% CI: 5.6%–9.5%; *P* = 0.004). Non-nucleoside reverse transcriptase inhibitors (NNRTIs)-associated resistance increased significantly over the study period (adjusted *P* = 0.009) driven primarily by K103N/S. In multivariable analysis, TDR was independently associated with recent infection (AOR = 1.66) and resident migrant status (AOR = 1.31). TDR sequences exhibited significant non-random clustering across transmission networks (*P* < 0.001), with 59.2% of networked TDR sequences concentrated in 2.7% of transmission clusters. Phylogeographic analyses indicated substantial cross-population linkages of TDR-associated lineages, with both temporary and resident migrants contributing disproportionately to TDR introduction. Resident migrants showed substantial network participation (44.1%), were overrepresented in TDR transmission clusters (78.9%), and disproportionately involved in TDR–TDR edges (74.3%), suggesting they may facilitate connectivity between non-local and local transmission networks. These findings support a structured pattern of TDR dissemination and highlight the role of population mobility in cross-population TDR dynamics, informing targeted surveillance in highly mobile urban settings.

## Introduction

The widespread implementation of antiretroviral therapy (ART) has transformed human immunodeficiency virus (HIV) infection into a manageable chronic condition, substantially reducing morbidity and mortality among people living with HIV [1]. However, sustained drug pressure has inevitably led to the emergence and onward transmission of drug-resistant HIV variants. These resistant strains can be transmitted to untreated individuals via drug resistance-associated mutations, leading to transmitted drug resistance (TDR), which directly compromises the effectiveness of first-line ART regimens as well as prevention strategies, including pre- and post-exposure prophylaxis [2–4]. Consequently, monitoring the prevalence of TDR has become a critical component of global HIV control programmes [5,6].

China faces complex TDR challenges due to its rapidly evolving HIV epidemic [7]. Nationwide TDR prevalence has risen progressively from 2.6% (2004–2007) to 7.8% (2002–2022), and further to 11.4% according to recent national surveillance in 2023 [7,8]. Notably, resistance to efavirenz/nevirapine (EFV/NVP) reached 6.5% in 2023 [8], approaching the 10% threshold at which the World Health Organization (WHO) recommends reconsideration of standard first-line antiretroviral regimens [5]. However, these estimates are largely based on newly diagnosed individuals and may not accurately reflect the current circulating burden of TDR, as “newly diagnosed” does not necessarily correspond to “recently infected”[9]. In the absence of drug pressure, some resistance mutations may revert to wild type due to associated fitness costs, becoming undetectable during chronic infection[10]. As a result, TDR prevalence is often higher among recently infected individuals and may better reflect currently circulating resistant strains [11]. Therefore, accurate assessment of TDR requires proper determination of infection recency. More importantly, given the dynamic nature of HIV transmission, prevalence estimates alone are insufficient to capture how resistant strains spread within and between populations. This underscores the need to move beyond static prevalence measures toward a deeper understanding of transmission dynamics.

Molecular transmission network analysis has been widely used to characterize clustering patterns and genetic linkages in HIV transmission, including the spread of drug-resistant strains [8,12]. By leveraging sequence similarity of HIV *pol* sequences, this approach reconstructs transmission clusters and captures the underlying network structure of the epidemic [13–15]. It has been increasingly applied in public health to identify transmission hotspots and highly-risk individuals, thereby informing targeted intervention strategies [16]. However, molecular transmission networks have limited ability to infer directional transmission or distinguish sources from recipients. Discrete phylogeographic models based on time-scaled phylogenetic trees provide a complementary probabilistic framework for reconstructing viral migration across populations or locations, enabling inference of major importation and exportation pathways [17]. These approaches have been widely applied to characterize spatial dissemination and migration-driven transmission dynamics of HIV, including CRF01_AE dissemination in the Philippines, subtype-specific epidemics in China, and migration-associated expansion of transmission networks in Europe [18–20]. While these methods offer complementary insights into transmission structure and directionality, they have rarely been jointly applied to investigate transmitted drug resistance.

Population mobility has long been recognized as a key driver of HIV transmission dynamics, particularly by linking otherwise distinct transmission networks across populations and geographic regions [21,22]. However, how population mobility influences the introduction, network connectivity, and onward dissemination of TDR remains poorly understood. As one of China’s largest metropolitan areas, Shanghai has maintained a comparatively low-level HIV epidemic, but its transmission landscape is characterized by predominantly sexual transmission and substantial population mobility. In the present citywide surveillance cohort, men who have sex with men (MSM) accounted for 56.0% of newly diagnosed infections, while resident migrants and temporary migrants represented 37.3% and 31.9% of participants, respectively. The epidemic was genetically diverse, with CRF07_BC and CRF01_AE representing the predominant circulating lineages (42.0% and 35.3%, respectively; Table 1). Together, these epidemiological and molecular features make Shanghai an informative setting in which to investigate the cross-population dissemination of drug-resistant HIV.

**Table 1.** Characteristics of individuals with newly diagnosed HIV-1 in Shanghai, 2018–2024.

Characteristics	Overall^1^	Temporary migrants^1^	Shanghai residents^1^	Resident migrants^1^	*P*
Total	7,538 (100.0%)	2,408 (31.9%)	2,319 (30.8%)	2,811 (37.3%)	
Age					**<0.001**^2^
18–29	2,454 (32.6%)	920 (38.2%)	371 (16.0%)	1,163 (41.4%)	
30–49	3,140 (41.7%)	914 (38.0%)	852 (36.7%)	1,374 (48.9%)	
≥50	1,944 (25.8%)	574 (23.8%)	1,096 (47.3%)	274 (9.7%)	
Gender					**<0**.**001**^2^
Men	6,876 (91.2%)	2,152 (89.4%)	2,101 (90.6%)	2,623 (93.3%)	
Women	662 (8.8%)	256 (10.6%)	218 (9.4%)	188 (6.7%)	
Ethnicity					**<0**.**001**^2^
Han	7,172 (95.1%)	2,273 (94.4%)	2,255 (97.2%)	2,644 (94.1%)	
Others	366 (4.9%)	135 (5.6%)	64 (2.8%)	167 (5.9%)	
Marital status					**<0**.**001**^2^
Divorced	840 (11.1%)	253 (10.5%)	349 (15.0%)	238 (8.5%)	
Married	2,535 (33.6%)	856 (35.5%)	1,003 (43.3%)	676 (24.0%)	
Single	4,047 (53.7%)	1,260 (52.3%)	926 (39.9%)	1,861 (66.2%)	
Unknown	116 (1.5%)	39 (1.6%)	41 (1.8%)	36 (1.3%)	
Education level					**<0**.**001**^2^
Primary or below	660 (8.8%)	320 (13.3%)	184 (7.9%)	156 (5.5%)	
Middle school	1,941 (25.7%)	794 (33.0%)	552 (23.8%)	595 (21.2%)	
High school	1,524 (20.2%)	552 (22.9%)	514 (22.2%)	458 (16.3%)	
College or above	3,389 (45.0%)	738 (30.6%)	1,063 (45.8%)	1,588 (56.5%)	
Unknown	24 (0.3%)	4 (0.2%)	6 (0.3%)	14 (0.5%)	
Transmission route					**<0**.**001^3^**
Heterosexual	3,080 (40.9%)	1,104 (45.8%)	1,162 (50.1%)	814 (29.0%)	
Injecting drug user	66 (0.9%)	23 (1.0%)	21 (0.9%)	22 (0.8%)	
MSM	4,220 (56.0%)	1,237 (51.4%)	1,085 (46.8%)	1,898 (67.5%)	
Others	12 (0.2%)	4 (0.2%)	3 (0.1%)	5 (0.2%)	
Unknown	160 (2.1%)	40 (1.7%)	48 (2.1%)	72 (2.6%)	
Infection recency					**<0**.**001**^2^
Chronic infection	5,744 (76.2%)	1,869 (77.6%)	1,807 (77.9%)	2,068 (73.6%)	
Recent infection	711 (9.4%)	27 (1.1%)	270 (11.6%)	414 (14.7%)	
Unknown	1,083 (14.4%)	512 (21.3%)	242 (10.4%)	329 (11.7%)	
Genotypes^4^					**<0**.**001**^2^
B	325 (4.3%)	116 (4.8%)	105 (4.5%)	104 (3.7%)	
CRF01_AE	2,664 (35.3%)	814 (33.8%)	819 (35.3%)	1,031 (36.7%)	
CRF07_BC	3,167 (42.0%)	990 (41.1%)	953 (41.1%)	1,224 (43.5%)	
CRF08_BC	262 (3.5%)	107 (4.4%)	115 (5.0%)	40 (1.4%)	
CRF55_01B	271 (3.6%)	95 (3.9%)	69 (3.0%)	107 (3.8%)	
CRF67_01B	90 (1.2%)	34 (1.4%)	28 (1.2%)	28 (1.0%)	
URF	417 (5.5%)	124 (5.1%)	113 (4.9%)	180 (6.4%)	
Others	342 (4.5%)	128 (5.3%)	117 (5.0%)	97 (3.5%)	

^1^ n (%) ^2^ χ² test; ^3^Simulated *P* values were used for transmission route due to small expected frequencies; ^4^Genotypes with an overall prevalence <1% were combined into the “Others” category.

In this study, we conducted a comprehensive analysis of newly diagnosed, ART-naïve HIV-infected individuals in Shanghai from 2018 to 2024. By integrating recent infection classification, genotypic resistance testing, molecular transmission network analysis, and time-scaled phylogeographic inference, we aimed to: (1) characterize prevalence and temporal trends in TDR; and (2) investigate how population mobility may shape the cross-population dissemination of drug-resistant HIV. Our findings reveal a structured, mobility-driven transmission pattern across population groups, providing a more mechanistic understanding of TDR dissemination and an evidence base for targeted resistance control strategies in highly mobile urban settings.

## Methods and materials

### Study population and design

Since 2018, specimens from individuals with confirmed HIV infection across Shanghai have been submitted to the Shanghai Municipal Center for Disease Control and Prevention (Shanghai CDC) for infection-recency and drug-resistance surveillance. Demographic and clinical information was obtained from the Shanghai CDC surveillance database. Between 2018 and 2024, 13,853 confirmed HIV cases were screened. After exclusion of duplicate records, individuals aged <18 years, and those with prior ART exposure, 12,838 unique ART-naïve adults were eligible for sequencing; 7,538 with quality-controlled HIV-1 *pol* sequences were included in the final analysis (Supplementary Figure 1).

Participants were categorized according to Shanghai household registration (hukou) status and recorded current residence. Individuals with Shanghai hukou were classified as Shanghai residents. Individuals without Shanghai hukou were classified as resident migrants if their recorded residence was within Shanghai and as temporary migrants if it was outside Shanghai. Foreign nationals were classified using the same residence-based criteria.

### Infection recency determination

Infection recency was initially assessed using the Limiting Antigen-Avidity Enzyme Immunoassay (LAg-Avidity EIA; Beijing Jinhao Biotechnology Co., Ltd.). To reduce false-recent classification, initial recency status was further evaluated using pretreatment viral load. Individuals initially classified as recently infected were reclassified as having chronic infection if pretreatment viral load was <1,000 copies/mL, retained as recently infected if viral load was ≥1,000 copies/mL, and classified as having unknown infection recency if no valid pretreatment viral load result was available.

### RNA extraction, sequencing, genotyping and drug resistance analysis

Viral RNA was extracted from plasma samples, followed by reverse transcription and nested PCR amplification of the HIV-1 *pol* (PR-RT) region. The integrase region was not included in the routine surveillance protocol during the study period and was therefore not analyzed. PCR products were sequenced using Sanger sequencing. All segments were assembled using the Sequencher v5.4.6 with reference to HXB2 *pol* segment. Ambiguous bases were considered when the secondary peak was more than 30% of the main peak. Sequences were aligned and trimmed to 1053 bp using Geneious Prime (version 2025.1.2).

A total of 7,591 HIV-1 *pol* sequences were obtained. Sequences containing >5% ambiguous nucleotides, duplicate sequences, or those flagged with a Stanford HIVDB quality assessment (QA) warning were excluded, leaving 7,538 sequences in the final analytical dataset (Supplementary Figure 1).

Genotypes were initially assigned using COMET and confirmed by maximum-likelihood phylogenetic analysis in IQ-TREE v1.6.12 under the GTR + G model. Branch support was assessed using 1,000 SH-aLRT and ultrafast bootstrap replicates; assignments with SH-aLRT ≥80% and UFBoot ≥95% were considered reliable [23]. Sequences not meeting these criteria were further evaluated for recombination using RIP 3.0.

Surveillance drug resistance mutations (SDRMs) in the PR–RT region were identified using the Stanford HIVDB Calibrated Population Resistance (CPR) tool, which screens HIV-1 sequence data against the WHO SDRM list to identify mutations associated with TDR. Individuals harboring ≥1 SDRM were classified as having TDR. Drug-class-specific TDR was assigned according to non-nucleoside reverse transcriptase inhibitor (NNRTI)-, nucleoside reverse transcriptase inhibitor (NRTI)-, or protease inhibitor (PI)-associated SDRMs.

### Molecular transmission network analysis

Pairwise genetic distances (GD) were calculated using the Tamura-Nei 93 (TN93) model implemented in veg/tn93. The primary mixed-genotype network was constructed using a GD threshold of ≤0.012 substitutions/site. TDR-containing clusters were defined as clusters containing ≥1 individual with TDR, whereas TDR transmission clusters contained ≥2 individuals with TDR [24]. Threshold sensitivity was evaluated at 0.005, 0.010, 0.012, and 0.015 substitutions/site, and optimal thresholds for the overall dataset and major HIV-1 genotypes were additionally estimated using AUTO-TUNE [25]. CRF07_BC networks were reconstructed across all four thresholds to assess threshold-dependent changes in network topology. Independent TDR transmission networks were reconstructed for CRF07_BC, CRF01_AE, and CRF55_01B (each n > 100) using their respective optimized thresholds. The robustness of resident migrant–associated network metrics across thresholds was assessed in the overall dataset and major TDR-associated genotypes. Network characteristics included cluster size, edges, and node degree; nodes with degree ≥5 were defined as high-degree nodes. Annual cumulative networks for 2018–2024 were reconstructed at the primary threshold to assess temporal network expansion. Networks were visualized in Cytoscape v3.10.4.

### Bayesian phylogeographic analysis

Bayesian phylogeographic analyses were performed using BEAST v1.10.4 with population groups treated as a discrete trait, using all TDR sequences within TDR transmission clusters (n = 77). The GTR + I + G substitution model was selected by ModelTest-NG V0.2.0, with an uncorrelated lognormal relaxed clock and a coalescent model with constant population size. Transmission linkages were inferred using Bayesian stochastic search variable selection (BSSVS). Three independent MCMC chains of 150 million generations were run for each analysis and combined in LogCombiner after removal of 10% burn-in. Convergence was confirmed by effective sample sizes (ESS) > 200 in Tracer v1.7.1.

To evaluate the robustness of this analysis, the same 77 sequences were down-sampled in ten independent year-stratified replicates based on the smallest population group, with equal numbers of sequences randomly sampled from the other groups by year without imputing missing years (Supplementary Table 1); each replicate was analyzed using the same BEAST workflow described above. Separate phylogeographic analyses were also performed for CRF07_BC and CRF01_AE, using the corresponding genotype-specific TDR sequences identified within TDR transmission clusters constructed at both the primary and genotype-specific optimal GD thresholds.

The maximum clade credibility (MCC) tree was generated using TreeAnnotator, and diffusion pathways, Bayes factors (BF), and posterior probabilities (PP) were calculated using SpreaD3 v0.9.7 [26]; with BF >3 considered statistically supported. MCC trees were visualized in iTOL v6.8, and supported transitions were visualized using the R package ggraph. ESS, BF, and PP estimates are provided in Supplementary Table 2.

### Statistical analysis

Categorical variables were compared via χ² /Fisher’s exact test, continuous variables via Kruskal–Wallis test, and temporal trends via Cochran – Armitage trend test. The Benjamini–Hochberg (BH) procedure was applied to control the false discovery rate in analyses involving multiple simultaneous comparisons; all other *P* values were unadjusted. BH-corrected values are reported as “adjusted *P*”. TDR prevalence estimates are reported with 95% confidence intervals calculated using the Wilson score method. To identify independent risk factors for TDR, all candidate variables were entered into a multivariable logistic regression model. A stepwise backward selection procedure based on AIC was applied (MASS package) to select the final model. Two – tailed *P* value < 0.05 was considered statistically significant. A permutation-based concentration analysis was performed by randomly shuffling TDR labels across clustered sequences (5,000 iterations) and comparing the observed cumulative distribution of TDR across clusters with the null distribution. All statistical analyses were performed using R (version 4.2.1).

### Ethics statement

This study was approved by the Ethics Committee of the Shanghai CDC (Approval No. 2021-13). As data were collected under routine public health surveillance, informed consent was not required.

## Results

### Characteristics of the study population

A total of 7,538 newly diagnosed HIV-1 infected individuals in Shanghai between 2018 and 2024 were included in this study (Table 1). Overall, the study population was predominantly male (91.2%) and of Han ethnicity (95.1%), with most participants aged 30–49 years (41.7%). Over half were unmarried (53.7%), and 45.0% had a college education or higher. MSM was the primary transmission route (56.0%).

By population group, 2,319 (30.8%) were Shanghai residents, 2,811 (37.3%) were resident migrants, and 2,408 (31.9%) were temporary migrants, with significant demographic and epidemiological differences (all *P* < 0.001). Resident migrants were younger, had the highest proportions of MSM transmission (67.5%) and recent infections (14.7%), along with higher educational attainment. In contrast, Shanghai residents were older (47.3% aged ≥50 years) and more likely to report heterosexual transmission (50.1%), while temporary migrants had lower education levels. Across all groups, CRF07_BC (42.0%) and CRF01_AE (35.3%) predominated. Migrant populations mainly originated from central-eastern and southwestern China, whereas temporary migrants were primarily residing in neighboring provinces (Table 1 and Supplementary Table 3).

### Prevalence and temporal trends of PR-RT TDR

**Figure 1.**
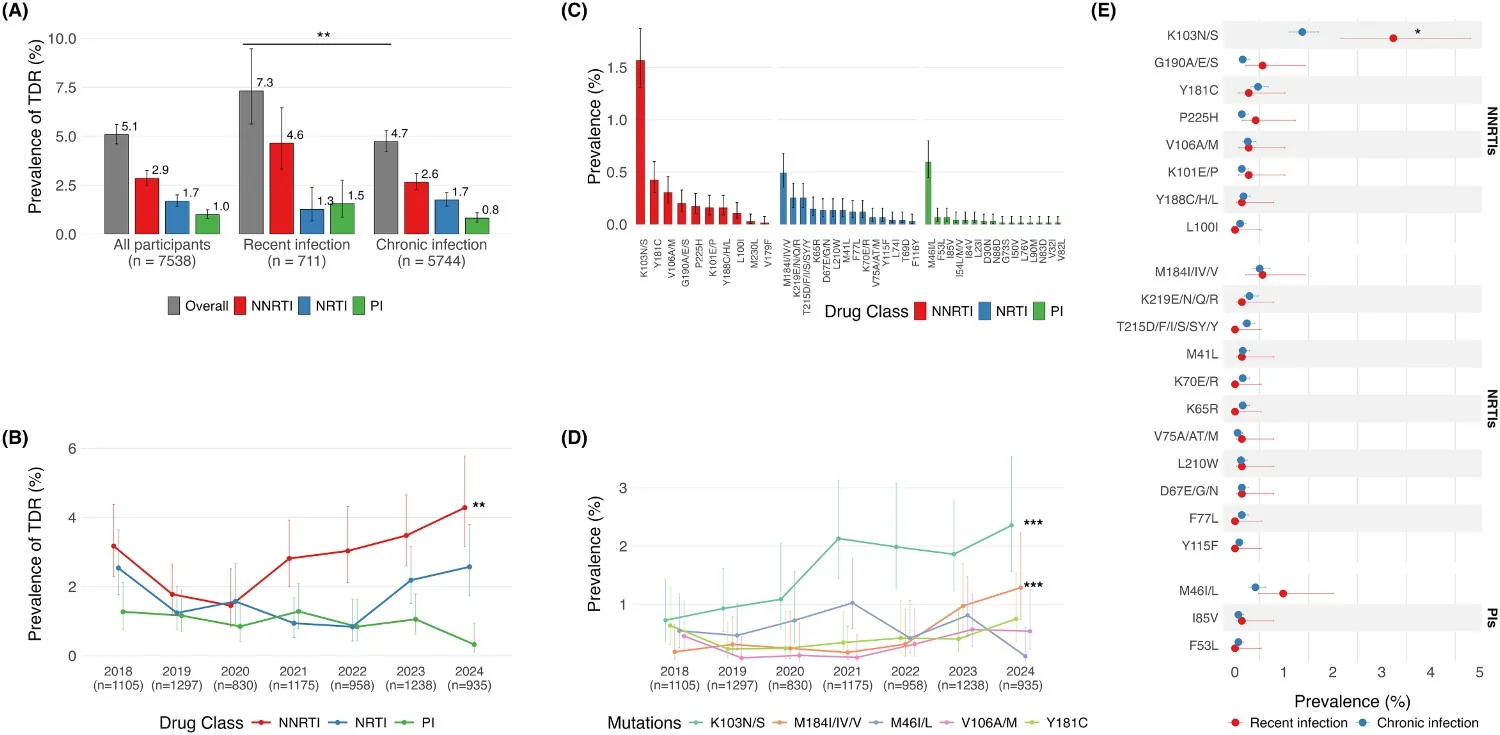
Prevalence and temporal trends of transmitted drug resistance (TDR) and major Surveillance Drug Resistance Mutations (SDRMs) in Shanghai, 2018–2024. (A) Prevalence of overall TDR and drug-class–specific resistance among all, recent, and chronic infections. (B) Annual prevalence of TDR by drug class. (C) Prevalence of major SDRMs. (D) Temporal trends of the five most prevalent SDRMs. (E) Prevalence of SDRMs stratified by recent and chronic infections; only mutations with prevalence ≥ 0.1% are shown. Asterisks indicate unadjusted *P* values in panel (A) and adjusted *P* values in panels (B), (D) and (E) (* *P* < 0.05, ** *P* < 0.01, *** *P* < 0.001). Error bars indicate 95% confidence intervals. NNRTIs: non-nucleoside reverse transcriptase inhibitors; NRTIs: nucleoside reverse transcriptase inhibitors; PIs: protease inhibitors.

The overall prevalence of TDR was 5.1% (95% CI: 4.6%–5.6%). The prevalence of TDR to NNRTIs, NRTIs, and PIs was 2.9% (95% CI: 2.5%–3.3%), 1.7% (95% CI: 1.4%–2.0%), and 1.0% (95% CI: 0.8%–1.2%), respectively (Figure 1A; exact prevalence estimates, 95% CIs, and sample sizes for Figure 1 are provided in Supplementary Table 4). Drug-specific resistance profiles (Supplementary Figure 2) showed that resistance was predominantly driven by NNRTI-associated drugs. From 2018 to 2024, annual TDR prevalence fluctuated across drug classes, with a significant increasing trend observed for NNRTI resistance (adjusted *P* = 0.009) (Figure 1B).

Participants were classified as having recent infection (n = 711), chronic infection (n = 5,744), or unknown infection recency (n = 1,083) based on the LAg-Avidity EIA combined with pretreatment viral load. TDR prevalence was significantly higher among individuals with recent infection than among those with chronic infection (7.3% [95% CI, 5.6%–9.5%] vs. 4.7% [95% CI, 4.2%–5.3%]; *P* = 0.004; Figure 1A).

### Distribution and temporal trends of SDRMs

At the SDRM level, the most prevalent NNRTI-associated mutation was K103N/S (1.6%), followed by Y181C (0.4%) and V106A/M (0.3%). Among NRTI-associated mutations, M184I/V was most common (0.5%), while M46I/L was the predominant PI-associated mutation (0.6%) (Figure 1C). Among the five major mutations, K103N/S and M184I/V exhibited significant increasing trends over time (adjusted *P* < 0.001 for both, Figure 1D). When stratified by infection recency, K103N/S was significantly more prevalent among individuals with recent infection than among those with chronic infection (3.2% vs. 1.4%; adjusted *P* = 0.014; Figure 1E).

### TDR prevalence by population group

**Figure 2.**
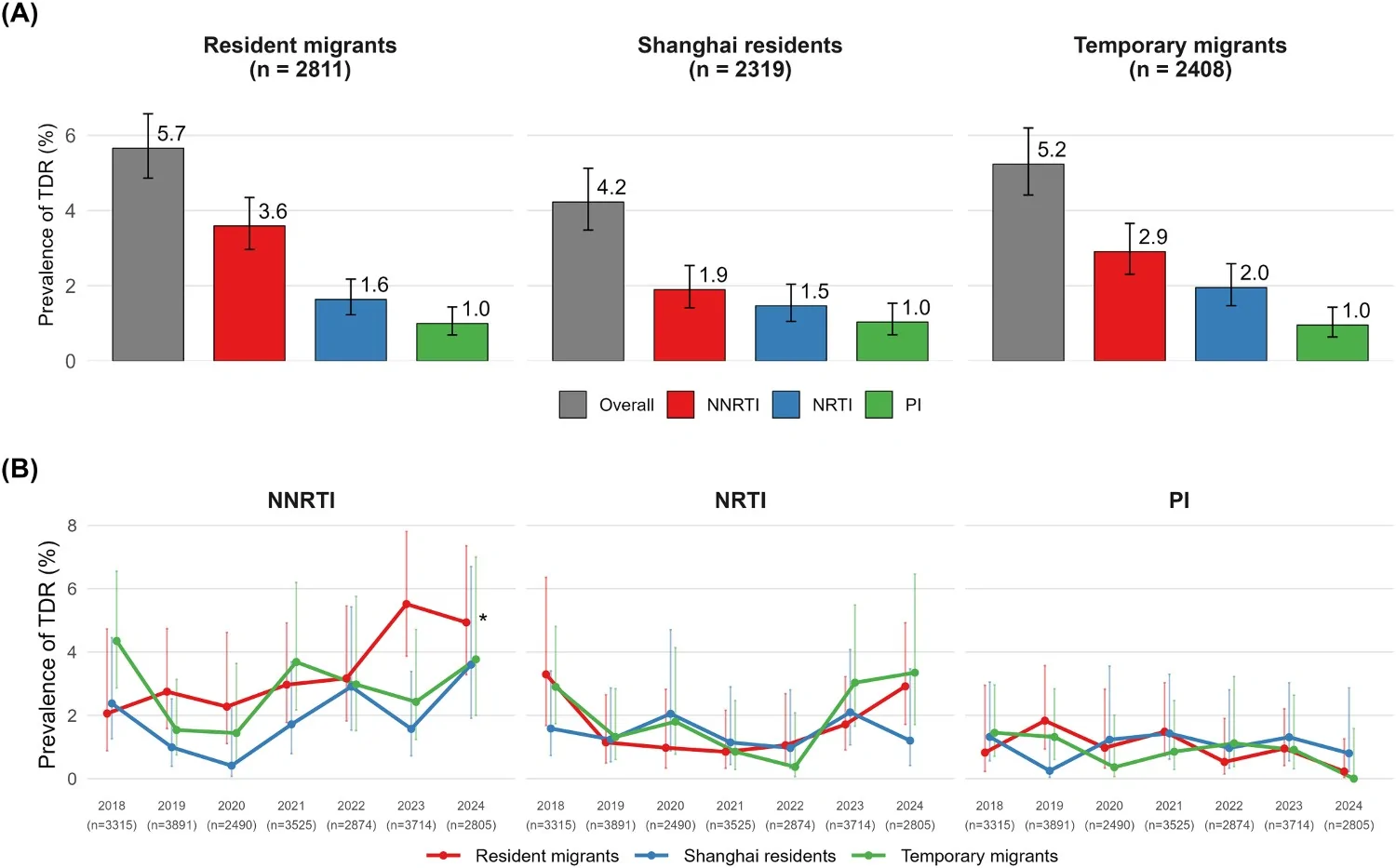
Transmitted Drug Resistance (TDR) prevalence across population groups. (A) Prevalence of overall and drug-class–specific TDR among resident migrants, Shanghai residents, and temporary migrants. Error bars indicate 95% confidence intervals. (B) Annual drug-class–specific TDR prevalence by population groups. Asterisks indicate adjusted *P* values (* adjusted *P* < 0.05).

Across population groups, TDR prevalence was highest among resident migrants (5.7%, 95% CI: 4.9%–6.6%), followed by temporary migrants (5.2%, 95% CI: 4.4%–6.2%) and Shanghai residents (4.2%, 95% CI: 3.5%–5.1%) (Figure 2A; exact values, 95% CIs, and sample sizes are provided in Supplementary Table 4). By drug class, NNRTI-associated TDR was highest among resident migrants (3.6%, 95% CI: 3.0%–4.3%), whereas temporary migrants showed relatively higher NRTI-associated TDR (2.0%, 95% CI: 1.5%–2.6%); PI-associated TDR was comparable across groups. Temporal analysis showed that a significant increase in NNRTI-associated TDR was observed only among resident migrants (adjusted *P* = 0.017) (Figure 2B). Together, these findings indicate that resident migrants had the highest TDR prevalence and the most pronounced increase in NNRTI-associated resistance among the three population groups.

### Factors associated with PR-RT TDR

Multivariable logistic regression identified several factors independently associated with TDR (Table 2). Resident migrants had higher odds of TDR compared with Shanghai residents (adjusted odds ratio [AOR] = 1.31; 95% CI: 1.00–1.70). Individuals infected through injecting drug use also showed increased odds compared with those infected through heterosexual contact (AOR = 2.91; 95% CI: 1.36–6.23). In addition, recent infection was associated with higher odds of TDR compared with chronic infection (AOR = 1.66; 95% CI: 1.20–2.29).

**Table 2.** Factors Associated with Transmitted Drug Resistance (TDR) Among Newly Diagnosed Individuals with HIV in Shanghai, 2018–2024.

Factors	Non-TDR (N = 7155)	TDR (N = 383)	OR (95% CI, *P*)	AOR (95% CI, *P*)
Population groups				
Shanghai residents	2221 (31.0%)	98 (25.6%)	Reference	Reference
Resident migrants	2652 (37.1%)	159 (41.5%)	**1.36 (1.05–1.76, *P*** **=** **0.020)**	**1.31 (1.00–1.70, *P*** **=** **0.047)**
Temporary migrants	2282 (31.9%)	126 (32.9%)	1.25 (0.95–1.64, *P* = 0.104)	1.27 (0.97–1.68, *P* = 0.086)
Transmission route				
Heterosexual	2942 (41.1%)	138 (36.0%)	Reference	Reference
Injecting drug user	58 (0.8%)	8 (2.1%)	**2.94 (1.38–6.28, *P*** **=** **0.005)**	**2.91 (1.36–6.23, *P*** **=** **0.006)**
MSM	3988 (55.7%)	232 (60.6%)	1.24 (1.00–1.54, *P* = 0.051)	1.20 (0.96–1.50, *P* = 0.113)
Others	12 (0.2%)	0 (0.0%)	0.00 (0.00-Inf, *P* = 0.964)	0.00 (0.00-Inf, *P* = 0.964)
Unknown	155 (2.2%)	5 (1.3%)	0.69 (0.28–1.70, *P* = 0.418)	0.50 (0.19–1.26, *P* = 0.142)
Recent infection status				
Chronic infection	5473 (76.5%)	271 (70.8%)	Reference	Reference
Recent infection	659 (9.2%)	52 (13.6%)	**1.59 (1.17–2.17, *P*** **=** **0.003)**	**1.66 (1.20–2.29, *P*** **=** **0.002)**
Unknown	1023 (14.3%)	60 (15.7%)	1.18 (0.89–1.58, *P* = 0.248)	1.16 (0.86–1.55, *P* = 0.330)
Baseline CD4 count, cells/µL				
<200	1942 (27.1%)	105 (27.4%)	Reference	Reference
200–349	2200 (30.7%)	104 (27.2%)	0.87 (0.66–1.15, *P* = 0.344)	0.81 (0.61–1.07, *P* = 0.133)
350–499	1452 (20.3%)	66 (17.2%)	0.84 (0.61–1.15, *P* = 0.281)	0.76 (0.55–1.05, *P* = 0.094)
≥500	888 (12.4%)	62 (16.2%)	1.29 (0.93–1.79, *P* = 0.122)	1.15 (0.83–1.60, *P* = 0.402)
Unknown	673 (9.4%)	46 (12.0%)	1.26 (0.88–1.81, *P* = 0.199)	1.39 (0.96–2.02, *P* = 0.079)

### Molecular transmission network

**Figure 3.**
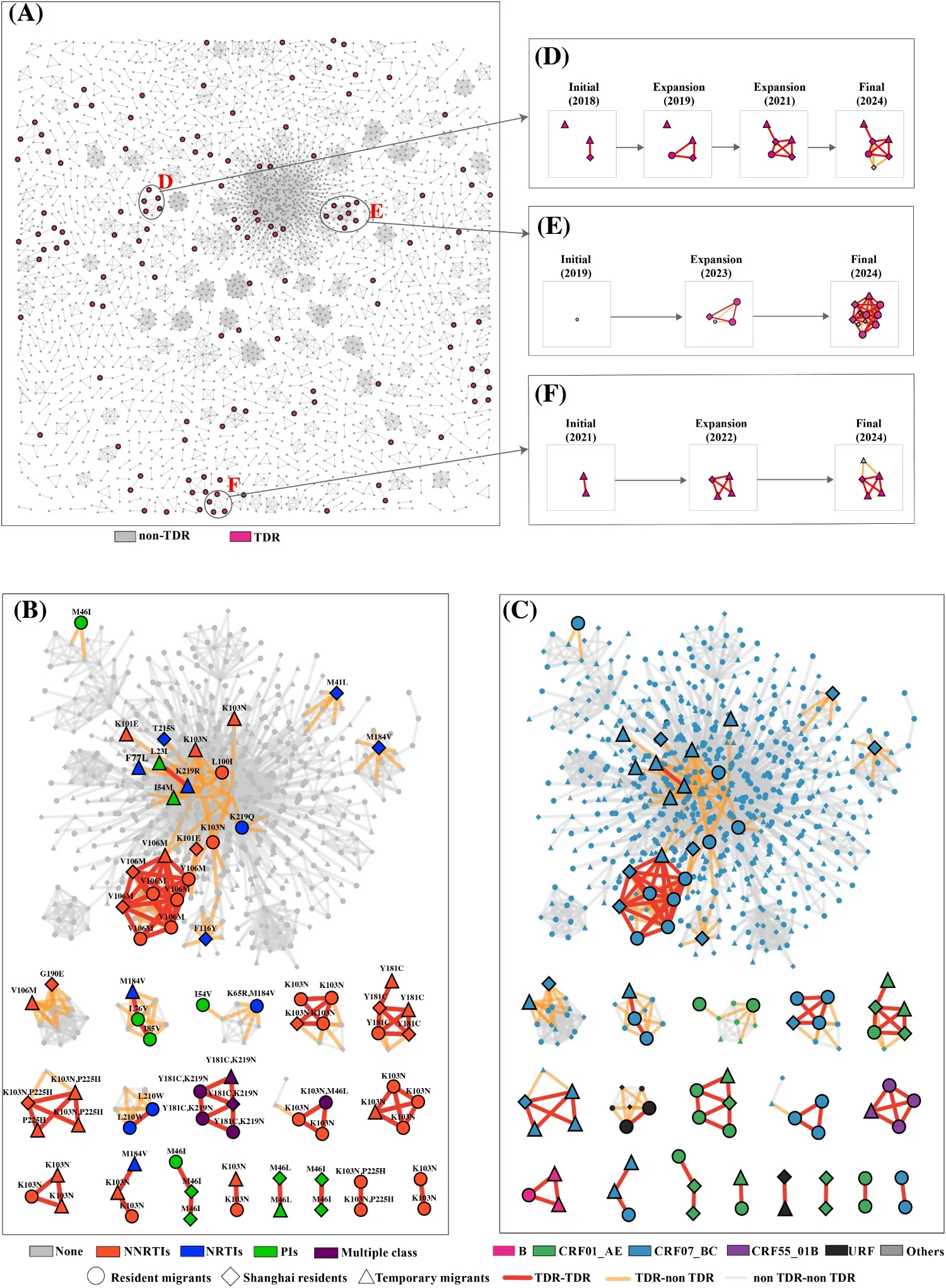
Molecular transmission network of HIV-1 in Shanghai, 2018–2024. (A) Overall network inferred using TN93 (≤0.012 substitutions/site). (B) TDR transmission clusters, defined as clusters containing ≥2 individuals with TDR. Resistance mutations are labeled above TDR nodes. (C) Same clusters as in (B) colored by HIV-1 genotype. (D–F) Representative mixed-population TDR transmission clusters showing the integration of different population groups over time.

At the primary GD threshold of 0.012 substitutions/site, which yielded the largest number of clusters in the mixed-genotype dataset (Supplementary Figure 3 and Supplementary Table 5), 3,132 individuals (41.5%) formed 695 transmission clusters ranging in size from 2 to 765 individuals (Figure 3A). The cumulative network expanded progressively from 2018 to 2024 (Supplementary Figure 4). Among the 695 clusters, 72 (10.3%) were TDR-containing clusters and included 130 individuals with TDR. TDR sequences were concentrated within a limited subset of clusters, and permutation analysis confirmed that this distribution was significantly non-random (*P* < 0.001).

We next examined whether network participation and connectivity differed across population groups. Network participation was highest among Shanghai residents (46.9%), followed by resident migrants (44.1%) and temporary migrants (33.4%) (χ² test, *P* < 0.001). Among clustered individuals, node degree also differed significantly across groups (Kruskal–Wallis test, *P* < 0.001). High-degree nodes (degree ≥5) accounted for 31.3% of Shanghai residents, 25.3% of resident migrants, and 23.8% of temporary migrants (χ² test, *P* < 0.001). At the edge level, 74 TDR–TDR edges (1.1%), 284 TDR–non-TDR edges (4.2%), and 6,393 non-TDR–non-TDR edges (94.7%) were identified. Of the 74 TDR–TDR edges, 55 (74.3%) involved at least one resident migrant (*P* = 0.002), and this pattern remained significant when the analysis was restricted to TDR transmission clusters (*P* = 0.024).

Because TDR sequences were concentrated within a small subset of the network, we further characterized clusters containing multiple individuals with TDR. Among the 72 TDR-containing clusters, 19 met the definition of TDR transmission clusters and contained 853 sequences, including 77 TDR sequences (Figure 3B). Although these clusters represented only 2.7% (19/695) of all transmission clusters, they contained 59.2% (77/130) of networked TDR sequences. TDR-positive resident migrants were present in 78.9% (15/19) of TDR transmission clusters. NNRTI-associated resistance occurred in 63.2% (12/19) of these clusters, with K103N (n = 28), Y181C (n = 10), M46I/L (n = 9), and V106M (n = 9) being the most frequent SDRMs. CRF07_BC and CRF01_AE predominated among TDR transmission clusters, and the largest cluster belonged to CRF07_BC (Figure 3C). Figure 3D–F illustrates the temporal expansion and changing population composition of three representative mixed-population TDR transmission clusters.

The predominance of CRF07_BC in the largest cluster raised the possibility that pooled-network topology and threshold selection could obscure genotype-specific transmission structures. We therefore compared CRF07_BC networks across four GD thresholds. Network topology varied markedly across thresholds, with progressive cluster merging at higher GD and reduced fine-scale resolution at the pooled threshold and above (Supplementary Figure 5). Genotype-specific TDR transmission clusters were subsequently reconstructed for CRF01_AE, CRF07_BC, and CRF55_01B using their respective optimized thresholds, revealing distinct SDRM profiles across genotypes (Supplementary Figure 6). In CRF01_AE, Y181C was the most frequent SDRM among TDR-positive individuals within TDR transmission clusters (13/34), followed by M46I/L (10/34) and K103N (8/34). In CRF07_BC, K103N predominated (15/38), followed by V106M (9/38), whereas all four TDR-positive individuals in the CRF55_01B TDR transmission cluster carried K103N. Dual-class resistance was also more frequent in CRF01_AE (8/34; seven NNRTI + NRTI and one NNRTI + PI) than in CRF07_BC (1/38; NNRTI + PI) and CRF55_01B (0/4).

Finally, we assessed whether the prominent representation of resident migrants in TDR-associated network structures depended on GD threshold or genotype. Across thresholds of 0.005–0.015 substitutions/site, TDR–TDR edges involving at least one resident migrant accounted for 74.0%–82.1% of such edges overall, 76.4%–100.0% in CRF07_BC, 63.2%–75.0% in CRF01_AE, and 100.0% in CRF55_01B. TDR transmission clusters involving TDR-positive resident migrants similarly accounted for 66.7%–81.0% overall, 75.0%–100.0% in CRF07_BC, 66.7%–87.5% in CRF01_AE, and 100.0% in CRF55_01B (Supplementary Table 5 and Supplementary Figure 7). Thus, although genotype and threshold selection affected fine-scale network topology and SDRM composition, the high representation of resident migrants within TDR-associated network structures persisted across the major circulating genotypes and tested thresholds.

### Phylogeographic inference of TDR dissemination

**Figure 4.**
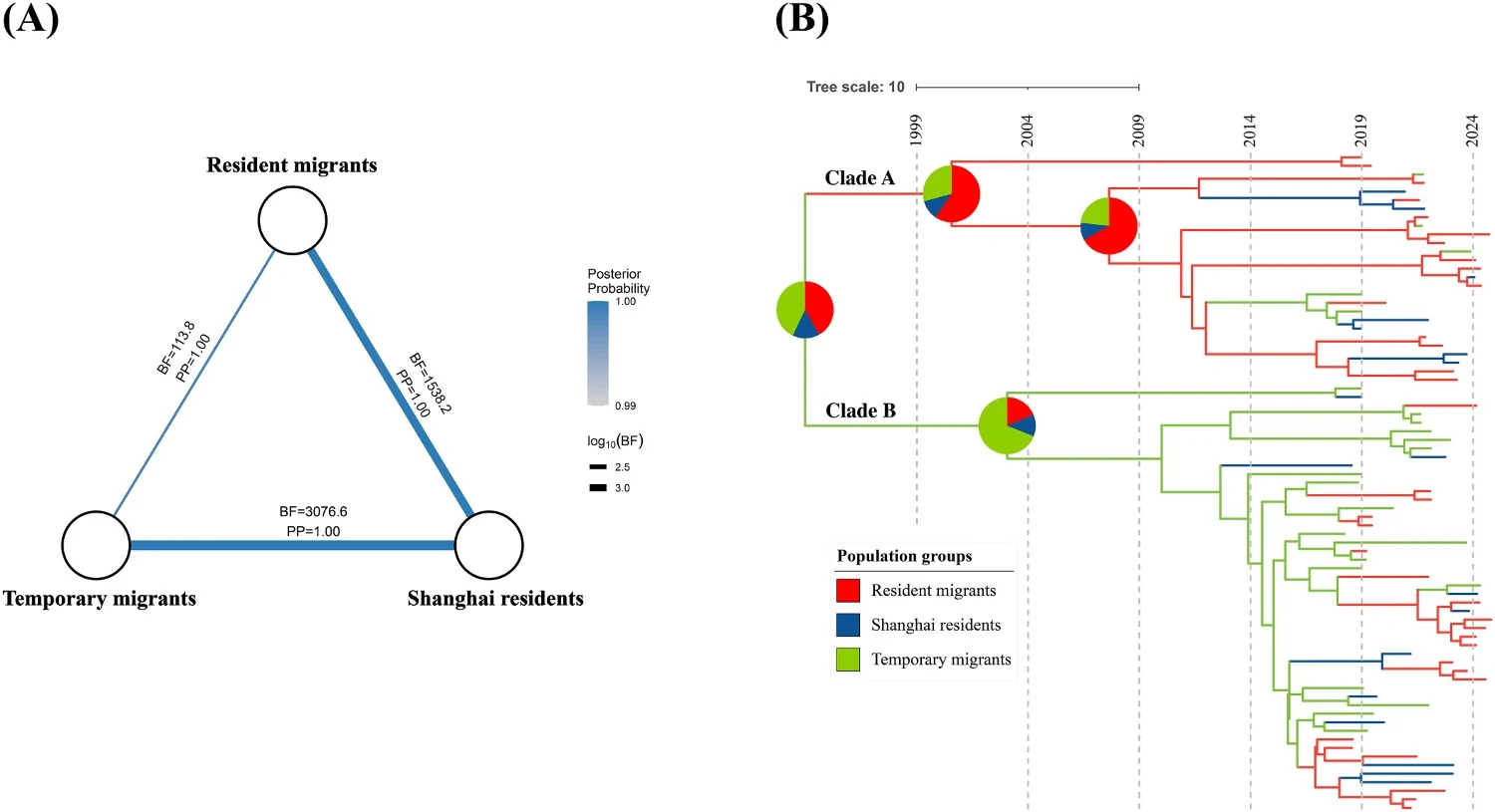
Phylogeographic linkages of transmitted drug resistance (TDR) among population groups. (A) Phylogeographic linkages inferred from 77 TDR sequences derived from TDR transmission clusters (≥2 individuals with TDR per cluster). Only linkages with Bayes factor (BF) > 3 are shown; corresponding posterior probabilities (PP) are indicated alongside each linkage. (B) Maximum clade credibility (MCC) tree of the 77 TDR sequences. Branch colors indicate inferred population groups, and pie charts represent posterior probabilities of inferred ancestral population states.

To further investigate TDR dissemination across population groups, we performed time-scaled discrete phylogeographic analyses using all TDR sequences within TDR transmission clusters (n = 77). BSSVS identified three statistically supported phylogeographic links (Figure 4A): temporary migrants and resident migrants (BF = 113.8), resident migrants and Shanghai residents (BF = 1538.2), and temporary migrants and Shanghai residents (BF = 3076.6), indicating substantial cross-population connectivity. Ten independent year-stratified down-sampling analyses consistently supported these major linkages despite variation in BF values (Supplementary Table 2).

Ancestral-state reconstruction further showed that inferred ancestral TDR lineages were predominantly associated with migrant populations, with 42.9% assigned to temporary migrants and 41.8% to resident migrants (Figure 4B). The overall MCC tree contained two major genotype-structured clades: Clade A was dominated by CRF01_AE (21/27, 77.8%), whereas Clade B was dominated by CRF07_BC (45/50, 90.0%). Because this strong genotype structure could influence ancestral-state inference in the pooled tree, we further performed genotype-specific phylogeographic analyses for CRF01_AE and CRF07_BC.

Within CRF01_AE, inferred ancestral TDR lineages were predominantly associated with resident migrants (74.4%; Supplementary Figure 8A), whereas within CRF07_BC they were predominantly associated with temporary migrants (67.3%; Supplementary Figure 8C). Similar ancestral-state patterns were recovered when the genotype-specific analyses were repeated using datasets defined by their respective optimized GD thresholds (Supplementary Figure 8B,D). These genotype-specific analyses therefore showed consistent support for phylogeographic linkages involving migrant populations and Shanghai residents across genotypes and GD thresholds (Supplementary Table 2), while also revealing genotype-dependent differences in the inferred population origins of TDR lineages .

## Discussion

In this study, we characterized TDR prevalence, clustering patterns, and cross-population dissemination using integrated epidemiological, molecular network, and phylogenetic approaches. Overall TDR prevalence in Shanghai remained at a moderate level, while NNRTI-associated resistance increased significantly over the study period. TDR sequences exhibited strong clustering within transmission networks, and phylogeographic analyses revealed structured cross-population linkages. Together, these findings highlight the role of population mobility in shaping the dynamics of drug-resistant HIV transmission in highly connected urban settings.

The overall TDR prevalence was 5.1%, corresponding to a moderate level (5%–15%) by WHO classification [5]. Compared with national estimates and higher-resistance provinces such as Hainan, Yunnan, and Xinjiang, Shanghai remained at a relatively lower level [8]. However, NNRTI-associated resistance increased significantly over time, whereas NRTI- and PI-associated resistance remained comparatively uncommon and showed no significant temporal increase. This pattern likely explains why overall TDR prevalence remained moderate despite the increasing NNRTI resistance burden. The increase in NNRTI-associated resistance was driven primarily by K103N/S, which was also the most frequently detected SDRM within TDR transmission clusters. K103N/S is known to impose a relatively limited fitness cost, allowing resistant variants to persist after transmission and continue circulating within established networks [24]. Its higher prevalence among individuals with recent infection further supports ongoing transmission of these variants. K103N/S also markedly reduces susceptibility to the NNRTIs nevirapine and efavirenz [27], underscoring its clinical relevance and the importance of continued resistance surveillance. In a highly connected metropolitan setting such as Shanghai, repeated introductions of resistant lineages through cross-regional population mobility may further contribute to the continued circulation of NNRTI-resistant variants.

The relationship between infection recency and individual resistance markers was more nuanced. Although M184 V/I imposes a substantial fitness cost and may revert toward wild type in the absence of drug pressure [24], its prevalence did not differ significantly between recent and chronic infections in our study (0.6% [4/711] vs. 0.5% [29/5,744]; adjusted *P* = 1.000; Supplementary Table 4E). This may reflect the cross-sectional nature of sampling at diagnosis, which cannot capture within-host reversion dynamics after transmission. Baseline CD4 count was likewise not associated with TDR in either univariate or multivariable logistic regression. Because CD4 count is influenced by substantial inter-individual variation in immune status and disease progression, it is an imprecise surrogate for infection recency, and any association between CD4 count and TDR mediated through recency would therefore be attenuated. Consistent with this, recency classified using the LAg-Avidity EIA together with pretreatment viral load, rather than CD4 count, remained independently associated with TDR.

Marked heterogeneity was observed across population groups. Resident migrants exhibited both the highest TDR prevalence and the most pronounced increase in NNRTI resistance, likely reflecting a higher proportion of younger age, MSM, and recent infections within this group [28]. Importantly, resident migrant status was independently associated with TDR in the multivariable logistic regression model, suggesting that this association cannot be fully explained by demographic composition or infection recency alone. Population mobility is also associated with poorer care engagement, facilitating both emergence and onward transmission of drug resistance [22]. Consistent with other high-mobility cities [29,30], resident migrants in Shanghai bear a disproportionate burden of TDR.

Molecular transmission network analysis further showed that TDR sequences were non-randomly concentrated within a small subset of transmission clusters, indicating that resistant variants were unevenly distributed across the local transmission network. When considered together with the phylogeographic analyses, distinct population-level patterns emerged. Migrant populations were more frequently associated with inferred ancestral TDR states, consistent with their disproportionate contribution to viral introduction; however, temporary migrants showed lower network participation and fewer high-degree nodes, suggesting a primarily source-like role rather than sustained onward transmission. In contrast, resident migrants showed enrichment across node-, cluster-, and edge-level network structures, consistent with a connecting role between non-local and local transmission networks. Shanghai residents, in turn, showed the highest overall network participation and the greatest proportion of high-degree nodes, consistent with a central role in sustaining local transmission. These complementary patterns suggest differentiated contributions of the three population groups to TDR transmission dynamics, with resident migrants potentially facilitating connectivity between non-local and local transmission networks. Importantly, the molecular network reflects genetic connectivity among individuals diagnosed through the Shanghai surveillance system and does not establish the geographic location or direction of individual transmission events.

Genotype-specific analyses further demonstrated heterogeneity in the population structure of TDR-associated lineages. In CRF07_BC, inferred ancestral lineages were more frequently associated with temporary migrants, whereas CRF01_AE lineages showed greater ancestral-state representation among resident migrants. Given the modest genotype-specific sample sizes, these differences should be interpreted cautiously. Importantly, genotype-specific network reconstruction using optimized GD thresholds showed that the high representation of resident migrants in TDR-associated edges and clusters persisted across the major circulating genotypes, despite substantial differences in network topology and SDRM composition. Together, these analyses indicate that the population-level patterns observed in the pooled network were unlikely to result solely from the CRF07_BC mega-cluster, threshold selection, or deep genotype divergence.

More broadly, these findings are consistent with evidence from other highly mobile Chinese cities showing that population mobility contributes to cross-population HIV connectivity [30,31], while further clarifying how distinct population groups contribute to the circulation of drug-resistant lineages within a single highly connected setting. They also suggest several population-specific opportunities for intervention. Baseline TDR screening among newly diagnosed migrant populations, together with improved cross-regional coordination, may facilitate early detection of resistant variants and timely linkage to appropriate treatment. For resident migrants, strengthening continuity of HIV care may reduce treatment interruptions and opportunities for onward dissemination of resistant virus. At the network level, molecular surveillance could be used to identify expanding TDR transmission clusters and guide targeted testing, prevention, and partner services. Together, these strategies address different aspects of TDR transmission dynamics in highly mobile urban settings.

## Limitations

Several limitations should be acknowledged. First, resistance analyses did not include integrase strand transfer inhibitor (INSTI) resistance and may therefore have slightly underestimated overall TDR prevalence. However, routine INSTI resistance surveillance conducted in Shanghai throughout 2025 identified only one integrase SDRM among 1,291 newly diagnosed individuals (0.08%), suggesting that this omission is unlikely to have materially altered the overall interpretation of our findings. Second, molecular transmission networks reflect genetic linkage rather than confirmed transmission events or directionality, while phylogeographic ancestral-state transitions indicate statistical associations between population groups rather than direct evidence of individual transmission events. Third, although pretreatment viral load was incorporated into the recency classification to reduce false-recent misclassification, residual uncertainty may remain because viral genotype and host-related factors can independently affect LAg-Avidity performance. Fourth, the relatively small number of TDR sequences limited the precision of ancestral-state reconstruction and phylogeographic inference. The same constraint precluded stable threshold optimization and network inference after further subdivision of CRF01_AE and CRF07_BC into individual sublineages; genotype-level stratification was therefore considered the most robust resolution supported by the present dataset. Fifth, temporary migrants may have acquired HIV outside Shanghai, potentially overestimating local transmission connectivity; however, they may also contribute to onward transmission during their stay, while their inclusion captures potentially important cross-regional genetic connectivity. The geographic location of individual linked transmission events cannot be determined from these data. Finally, because Shanghai has distinctive demographic and mobility characteristics, the generalizability of these findings to other settings may be limited.

## Conclusions

In conclusion, our findings indicate that population mobility is closely associated with the cross-population circulation of HIV-1 TDR in Shanghai. The combined molecular network and phylogeographic analyses support a structured pattern in which migrant populations were strongly represented in inferred ancestral states, resident migrants may facilitate connectivity between non-local and local transmission networks, and Shanghai residents showed greater connectivity within established local networks. These findings provide a framework for understanding how drug-resistant HIV circulates across highly mobile populations and support integrated strategies combining early resistance detection, continuity of care, molecular surveillance, and targeted intervention in metropolitan settings.

## Supplementary Material

00_260818_Supplementary_Tables.docx

00_260817_Supplementary_Figures.docx
