# Supplementary material for: HIV-1 transmitted drug resistance in Shanghai, 2018–2024: population mobility shapes cross-population TDR transmission dynamics
Source: Emerg Microbes Infect. 2026 Sep 18;15(1):2731504. doi: 10.1080/22221751.2026.2731504 (PMC13592006; doi:10.1080/22221751.2026.2731504)
Supplement: Supplementary file 1 [file TEMI_A_2731504_SM4935.docx]

**Supplementary Figures**

**Supplementary Figure 1**

**Supplementary Figure 1.** Flowchart of participant selection and sequence quality control (2018–2024).

**Supplementary Figure****2**

**Supplementary Figure 2.** Profiles Differences in HIV Drug Resistance to Antiretroviral Drugs Among Newly Diagnosed Individuals in Shanghai, 2018–2024.

ABC, abacavir; AZT, azidothymidine; D4T, stavudine; DDI, didanosine; FTC, emtricitabine; 3TC, lamivudine; TDF, tenofovir disoproxil fumarate; DOR, doravirine; EFV, efavirenz; ETV, etravirine; NVP, nevirapine; RPV, rilpivirine; ATV, atazanavir; DRV, darunavir; FPV, fosamprenavir; IDV, indinavir; LPV, lopinavir; NFV, nelfinavir; SQV, saquinavir; TPV, tipranavir.

**Supplementary Figure 3**

**Supplementary Figure 3.** Sensitivity analysis of molecular transmission network characteristics across a range of TN93 genetic distance thresholds for the overall dataset and major HIV-1 genotypes.

The black line and points indicate the number of transmission clusters identified at each threshold; blue line and points represent the percentage of sequences in clusters. Red dashed vertical lines denote genetic distance thresholds recommended by the AUTO-TUNE. The left y-axis corresponds to the number of clusters, and the right y-axis corresponds to the proportion of clustered sequences.

**Supplementary Figure 4**

**Supplementary Figure 4** Annual expansion dynamics of HIV molecular transmission networks inferred using a TN93 genetic distance threshold of 0.012, 2018–2024.

(A) Longitudinal trends of normalized network metrics.

(B–H) Corresponding network topology visualizations for each cumulative year from 2018 to 2024.

**Supplementary Figure 5**

**Supplementary Figure 5.** Sensitivity analysis of CRF07_BC molecular transmission networks across TN93 genetic distance (GD) thresholds.

(A) GD = 0.005. (B) GD = 0.010. (C) GD = 0.012. (D) GD = 0.015.

**Supplementary Figure 6**

**Supplementary Figure 6.** Genotype-specific TDR transmission clusters containing ≥2 TDR sequences inferred using genotype-specific optimal genetic distance (GD) thresholds.

(A) CRF01_AE (GD = 0.015). (B) CRF07_BC (GD = 0.010). (C) CRF55_01B (GD = 0.012).

**Supplementary Figure 7**

**Supplementary Figure 7.** Robustness of Resident migrant–Related Network Metrics Across Different Genetic Distance Thresholds.

Exact values underlying these metrics are provided in Supplementary Table 5.

**Supplementary Figure 8**

**Supplementary Figure 8.** Sensitivity analysis of Bayesian phylogeographic inference using the original and subtype-specific optimal genetic distance (GD) thresholds.

Maximum clade credibility (MCC) trees of CRF01_AE (A, GD=0.012; B, GD=0.015) and CRF07_BC (C, GD=0.012; D, GD=0.010). Pie charts indicate posterior probabilities of inferred ancestral population states.
