# Supplementary material for: HIV-1 transmitted drug resistance in Shanghai, 2018–2024: population mobility shapes cross-population TDR transmission dynamics
Source: Emerg Microbes Infect. 2026 Sep 18;15(1):2731504. doi: 10.1080/22221751.2026.2731504 (PMC13592006; doi:10.1080/22221751.2026.2731504)
Supplement: Supplementary file 2 [file TEMI_A_2731504_SM4936.docx]

| **Supplementary Table 1. Comparison of the original and down-sampled datasets by calendar year and population group.** | | | | | | | |
| --- | --- | --- | --- | --- | --- | --- | --- |
| **Year** | **RM (Before)** | **TM (Before)** | **SR (Before)** | **RM (After)** | **TM (After)** | **SR (After)** |  |
| **2018** | 3 | 8 | 3 | 3 | 3 | 3 |  |
| **2019** | 5 | 3 | 2 | 2 | 2 | 2 |  |
| **2020** | 0 | 1 | 1 | 0 | 1 | 1 |  |
| **2021** | 10 | 7 | 4 | 4 | 4 | 4 |  |
| **2022** | 5 | 1 | 3 | 3 | 1 | 3 |  |
| **2023** | 6 | 2 | 4 | 4 | 2 | 4 |  |
| **2024** | 7 | 1 | 1 | 1 | 1 | 1 |  |
| **Total** | **36** | **23** | **18** | **17** | **14** | **18** |  |
| *RM: resident migrants; TM: temporary migrants; SR: Shanghai residents. Shanghai residents were fully retained in all replicates (serving as the fixed year-stratified sampling quota, based on their smallest total sample size among the three groups), whereas resident migrants and temporary migrants were independently subsampled in each of the ten replicates; although the year-stratified sampling quota was therefore identical across replicates, the specific sequences sampled differed in each replicate (see Methods).* | | | | | | | |

| **Supplementary Table 2. Summary of Bayesian phylogeographic analyses, including minimum effective sample sizes (ESS), Bayes factors (BF), and posterior probabilities (PP) for supported phylogeographic linkages.** | | | | | |
| --- | --- | --- | --- | --- | --- |
| **Dataset** | **Minimum**  **ESS** | **TM–RM BF (PP)** | **TM–SR BF (PP)** | **RM–SR BF (PP)** |  |
| ***Overall analysis*** | | | | | |
| **Overall (n=77)** | 1336 | 113.8 (1.00) | 3076.6 (1.00) | 1538.2 (1.00) |  |
| ***Genotype-specific analyses*** | | | | | |
| **CRF07_BC (GD=0.012, n=45)** | 831 | 5.3 (0.98) | 205.0 (1.00) | 4.0 (0.97) |  |
| **CRF07_BC (GD=0.010, n=38)** | 3479 | — | 307.6 (1.00) | 4.3 (0.97) |  |
| **CRF01_AE (GD=0.012, n=21)** | 1001 | — | 31.9 (1.00) | 73.1 (1.00) |  |
| **CRF01_AE (GD=0.015, n=34)** | 3421 | — | 253.7 (1.00) | 3045.8 (1.00) |  |
| ***Down-sampling analyses*** | | | | | |
| **Random 1 (n=49)** | 1201 | 11.9 (0.99) | 9.0 (0.99) | 2922.7 (1.00) |  |
| **Random 2 (n=49)** | 1117 | 49.5 (1.00) | 90.4 (1.00) | 53.9 (1.00) |  |
| **Random 3 (n=49)** | 1297 | 28.6 (1.00) | 161.8 (1.00) | 16.7 (0.99) |  |
| **Random 4 (n=49)** | 1254 | 512.7 (1.00) | 3.7 (0.97) | 25.7 (1.00) |  |
| **Random 5 (n=49)** | 1844 | — | 34.8 (1.00) | 279.6 (1.00) |  |
| **Random 6 (n=49)** | 1912 | 123.0 (1.00) | — | 113.8 (1.00) |  |
| **Random 7 (n=49)** | 1498 | 9.8 (0.99) | 769.1 (1.00) | 6.4 (0.98) |  |
| **Random 8 (n=49)** | 1921 | 9.2 (0.99) | 2953.5 (1.00) | 42.7 (1.00) |  |
| **Random 9 (n=49)** | 2487 | 5.5 (0.98) | — | 118.2 (1.00) |  |
| **Random 10 (n=49)** | 2303 | 507.5 (1.00) | 4.9 (0.98) | 1015.2 (1.00) |  |
| *GD: genetic distance; TM: temporary migrants; RM: resident migrants; SR: Shanghai residents. Only transitions with BF > 3 are shown. All datasets were analyzed using three independent MCMC chains of 150 million generations each. Minimum ESS represents the lowest effective sample size among all estimated parameters in the combined log file for each dataset.* | | | | | |

**Supplementary Table 3. Characteristics of Individuals with Newly Diagnosed HIV-1 in Shanghai, 2018–2024 (extended characteristics).**

| **Characteristic** | **Overall** | **Temporary**  **migrants** | **Shanghai**  **residents** | **Resident**  **migrants** | **P** |
| --- | --- | --- | --- | --- | --- |
| **Year** |  |  |  |  | **<0.001** |
| 2018 | 1105 (14.7%) | 483 (20.1%) | 379 (16.3%) | 243 (8.6%) |  |
| 2019 | 1297 (17.2%) | 456 (18.9%) | 404 (17.4%) | 437 (15.5%) |  |
| 2020 | 830 (11.0%) | 278 (11.5%) | 244 (10.5%) | 308 (11.0%) |  |
| 2021 | 1175 (15.6%) | 353 (14.7%) | 350 (15.1%) | 472 (16.8%) |  |
| 2022 | 958 (12.7%) | 269 (11.2%) | 310 (13.4%) | 379 (13.5%) |  |
| 2023 | 1238 (16.4%) | 330 (13.7%) | 382 (16.5%) | 526 (18.7%) |  |
| 2024 | 935 (12.4%) | 239 (9.9%) | 250 (10.8%) | 446 (15.9%) |  |
| **Occupation** |  |  |  |  | **<0.001** |
| Business services | 2448 (32.5%) | 756 (31.4%) | 536 (23.1%) | 1156 (41.1%) |  |
| Farmers or fishers | 295 (3.9%) | 162 (6.7%) | 87 (3.8%) | 46 (1.6%) |  |
| Office staff | 572 (7.6%) | 113 (4.7%) | 209 (9.0%) | 250 (8.9%) |  |
| Retired | 784 (10.4%) | 147 (6.1%) | 587 (25.3%) | 50 (1.8%) |  |
| Students | 191 (2.5%) | 49 (2.0%) | 50 (2.2%) | 92 (3.3%) |  |
| Teachers | 59 (0.8%) | 9 (0.4%) | 31 (1.3%) | 19 (0.7%) |  |
| Workers | 745 (9.9%) | 286 (11.9%) | 156 (6.7%) | 303 (10.8%) |  |
| Unemployed | 1284 (17.0%) | 536 (22.3%) | 335 (14.4%) | 413 (14.7%) |  |
| Others | 507 (6.7%) | 157 (6.5%) | 128 (5.5%) | 222 (7.9%) |  |
| Unknown | 653 (8.7%) | 193 (8.0%) | 200 (8.6%) | 260 (9.2%) |  |
| **STD history** |  |  |  |  | **<0.001** |
| No | 4469 (59.3%) | 1487 (61.8%) | 1300 (56.1%) | 1682 (59.8%) |  |
| Yes | 1285 (17.0%) | 412 (17.1%) | 379 (16.3%) | 494 (17.6%) |  |
| Unknown | 1784 (23.7%) | 509 (21.1%) | 640 (27.6%) | 635 (22.6%) |  |
| **Baseline CD4 count** |  |  |  |  | **<0.001** |
| <200 | 2047 (27.2%) | 660 (27.4%) | 742 (32.0%) | 645 (22.9%) |  |
| 200–349 | 2304 (30.6%) | 715 (29.7%) | 673 (29.0%) | 916 (32.6%) |  |
| 350–499 | 1518 (20.1%) | 437 (18.1%) | 455 (19.6%) | 626 (22.3%) |  |
| ≥500 | 950 (12.6%) | 305 (12.7%) | 264 (11.4%) | 381 (13.6%) |  |
| Unknown | 719 (9.5%) | 291 (12.1%) | 185 (8.0%) | 243 (8.6%) |  |
| **Source of sample** |  |  |  |  | **<0.001** |
| VCT | 2675 (35.5%) | 1245 (44.3%) | 779 (32.4%) | 651 (28.1%) |  |
| Hospital inpatient/outpatient settings | 2205 (29.3%) | 643 (22.9%) | 733 (30.4%) | 829 (35.7%) |  |
| Preoperative screening | 1113 (14.8%) | 273 (9.7%) | 416 (17.3%) | 424 (18.3%) |  |
| Dermatology/urology STD clinics | 1063 (14.1%) | 484 (17.2%) | 307 (12.7%) | 272 (11.7%) |  |
| Detainee testing | 203 (2.7%) | 70 (2.5%) | 99 (4.1%) | 34 (1.5%) |  |
| Pre-transfusion screening | 99 (1.3%) | 34 (1.2%) | 20 (0.8%) | 45 (1.9%) |  |
| Others | 180 (2.4%) | 62 (2.2%) | 54 (2.2%) | 64 (2.8%) |  |

*Values are n (%) unless otherwise indicated. P values were calculated using Pearson's chi-squared test.*

**Supplementary Table 4A. Prevalence of transmitted drug resistance (TDR) overall and by drug class, stratified by infection recency status (corresponds to Figure 1A).**

| **Category** | **N** | **Overall TDR,**  **n (%, 95% CI)** | **NNRTI, n (%, 95% CI)** | **NRTI, n (%, 95% CI)** | **PI, n (%, 95% CI)** |
| --- | --- | --- | --- | --- | --- |
| **All participants** | 7538 | 383 (5.1%, 4.6–5.6%) | 215 (2.9%, 2.5–3.3%) | 127 (1.7%, 1.4–2.0%) | 75 (1.0%, 0.8–1.2%) |
| **Recent infection** | 711 | 52 (7.3%, 5.6–9.5%) | 33 (4.6%, 3.3–6.4%) | 9 (1.3%, 0.7–2.4%) | 11 (1.5%, 0.9–2.7%) |
| **Chronic infection** | 5744 | 271 (4.7%, 4.2–5.3%) | 152 (2.6%, 2.3–3.1%) | 100 (1.7%, 1.4–2.1%) | 47 (0.8%, 0.6–1.1%) |

**Supplementary Table 4B. Annual prevalence of transmitted drug resistance (TDR) by drug class, 2018–2024 (corresponds to Figure 1B).**

| **Year** | **N** | **NNRTI, n (%, 95% CI)** | **NRTI, n (%, 95% CI)** | **PI, n (%, 95% CI)** |
| --- | --- | --- | --- | --- |
| **2018** | 1105 | 35 (3.2%, 2.3–4.4%) | 28 (2.5%, 1.8–3.6%) | 14 (1.3%, 0.8–2.1%) |
| **2019** | 1297 | 23 (1.8%, 1.2–2.6%) | 16 (1.2%, 0.8–2.0%) | 15 (1.2%, 0.7–1.9%) |
| **2020** | 830 | 12 (1.4%, 0.8–2.5%) | 13 (1.6%, 0.9–2.7%) | 7 (0.8%, 0.4–1.7%) |
| **2021** | 1175 | 33 (2.8%, 2.0–3.9%) | 11 (0.9%, 0.5–1.7%) | 15 (1.3%, 0.8–2.1%) |
| **2022** | 958 | 29 (3.0%, 2.1–4.3%) | 8 (0.8%, 0.4–1.6%) | 8 (0.8%, 0.4–1.6%) |
| **2023** | 1238 | 43 (3.5%, 2.6–4.6%) | 27 (2.2%, 1.5–3.2%) | 13 (1.1%, 0.6–1.8%) |
| **2024** | 935 | 40 (4.3%, 3.2–5.8%) | 24 (2.6%, 1.7–3.8%) | 3 (0.3%, 0.1–0.9%) |

**Supplementary Table 4C. Prevalence of individual surveillance drug resistance mutations (SDRMs)**

**among all participants (N = 7,538) (corresponds to Figure 1C).**

| **Drug class** | **Mutation** | **n** | **Prevalence, % (95% CI)** |
| --- | --- | --- | --- |
| **NNRTI** | K103N/S | 118 | 1.57%, 1.31–1.87% |
|  | Y181C | 32 | 0.42%, 0.30–0.60% |
|  | V106A/M | 23 | 0.31%, 0.20–0.46% |
|  | G190A/E/S | 15 | 0.20%, 0.12–0.33% |
|  | P225H | 13 | 0.17%, 0.10–0.29% |
|  | K101E/P | 12 | 0.16%, 0.09–0.28% |
|  | Y188C/H/L | 12 | 0.16%, 0.09–0.28% |
|  | L100I | 8 | 0.11%, 0.05–0.21% |
|  | M230L | 2 | 0.03%, 0.01–0.10% |
|  | V179F | 1 | 0.01%, 0.00–0.08% |
| **NRTI** | M184I/IV/V | 37 | 0.49%, 0.36–0.68% |
|  | K219E/N/Q/R | 19 | 0.25%, 0.16–0.39% |
|  | T215D/F/I/S/SY/Y | 19 | 0.25%, 0.16–0.39% |
|  | K65R | 11 | 0.15%, 0.08–0.26% |
|  | D67E/G/N | 10 | 0.13%, 0.07–0.24% |
|  | L210W | 10 | 0.13%, 0.07–0.24% |
|  | M41L | 10 | 0.13%, 0.07–0.24% |
|  | F77L | 9 | 0.12%, 0.06–0.23% |
|  | K70E/R | 9 | 0.12%, 0.06–0.23% |
|  | V75A/AT/M | 5 | 0.07%, 0.03–0.16% |
|  | Y115F | 5 | 0.07%, 0.03–0.16% |
|  | L74I | 3 | 0.04%, 0.01–0.12% |
|  | T69D | 3 | 0.04%, 0.01–0.12% |
|  | F116Y | 2 | 0.03%, 0.01–0.10% |
| **PI** | M46I/L | 45 | 0.60%, 0.45–0.80% |
|  | F53L | 5 | 0.07%, 0.03–0.16% |
|  | I85V | 5 | 0.07%, 0.03–0.16% |
|  | I54L/M/V | 3 | 0.04%, 0.01–0.12% |
|  | I84V | 3 | 0.04%, 0.01–0.12% |
|  | L23I | 3 | 0.04%, 0.01–0.12% |
|  | D30N | 2 | 0.03%, 0.01–0.10% |
|  | N88D | 2 | 0.03%, 0.01–0.10% |
|  | G73S | 1 | 0.01%, 0.00–0.08% |
|  | I50V | 1 | 0.01%, 0.00–0.08% |
|  | L76V | 1 | 0.01%, 0.00–0.08% |
|  | L90M | 1 | 0.01%, 0.00–0.08% |
|  | N83D | 1 | 0.01%, 0.00–0.08% |
|  | V32I | 1 | 0.01%, 0.00–0.08% |
|  | V82L | 1 | 0.01%, 0.00–0.08% |

**Supplementary Table 4D. Annual prevalence of the five most common surveillance drug resistance mutations (SDRMs), 2018–2024 (corresponds to Figure 1D).**

| **Year** | **N** | **K103N/S,**  **n (%, 95% CI)** | **M184I/IV/V,**  **n (%, 95% CI)** | **M46I/L,**  **n (%, 95% CI)** | **V106A/M,**  **n (%, 95% CI)** | **Y181C,**  **n (%, 95% CI)** |
| --- | --- | --- | --- | --- | --- | --- |
| **2018** | 1105 | 8 (0.72%, 0.37–1.42%) | 2 (0.18%, 0.05–0.66%) | 6 (0.54%, 0.25–1.18%) | 5 (0.45%, 0.19–1.05%) | 7 (0.63%, 0.31–1.30%) |
| **2019** | 1297 | 12 (0.93%, 0.53–1.61%) | 4 (0.31%, 0.12–0.79%) | 6 (0.46%, 0.21–1.01%) | 1 (0.08%, 0.01–0.44%) | 3 (0.23%, 0.08–0.68%) |
| **2020** | 830 | 9 (1.08%, 0.57–2.05%) | 2 (0.24%, 0.07–0.87%) | 6 (0.72%, 0.33–1.57%) | 1 (0.12%, 0.02–0.68%) | 2 (0.24%, 0.07–0.87%) |
| **2021** | 1175 | 25 (2.13%, 1.45–3.12%) | 2 (0.17%, 0.05–0.62%) | 12 (1.02%, 0.59–1.78%) | 1 (0.09%, 0.02–0.48%) | 4 (0.34%, 0.13–0.87%) |
| **2022** | 958 | 19 (1.98%, 1.27–3.08%) | 3 (0.31%, 0.11–0.92%) | 4 (0.42%, 0.16–1.07%) | 3 (0.31%, 0.11–0.92%) | 4 (0.42%, 0.16–1.07%) |
| **2023** | 1238 | 23 (1.86%, 1.24–2.77%) | 12 (0.97%, 0.56–1.69%) | 10 (0.81%, 0.44–1.48%) | 7 (0.57%, 0.27–1.16%) | 5 (0.40%, 0.17–0.94%) |
| **2024** | 935 | 22 (2.35%, 1.56–3.54%) | 12 (1.28%, 0.74–2.23%) | 1 (0.11%, 0.02–0.60%) | 5 (0.53%, 0.23–1.25%) | 7 (0.75%, 0.36–1.54%) |

**Supplementary Table 4E. Prevalence of individual surveillance drug resistance mutations (SDRMs) among recently infected versus chronically infected individuals, with Benjamini–Hochberg–adjusted P values (corresponds to Figure 1E).**

| **Drug class** | **Mutation** | **Overall, n (%)** | **Recent infection,**  **n (%, 95% CI)** | **Chronic infection,**  **n (%, 95% CI)** | **Adjusted P** |
| --- | --- | --- | --- | --- | --- |
| **NNRTI** | K103N/S | 102 (1.6%) | 23 (3.23%, 2.17–4.81%) | 79 (1.38%, 1.11–1.71%) | 0.014 |
|  | Y181C | 29 (0.4%) | 2 (0.28%, 0.08–1.02%) | 27 (0.47%, 0.32–0.68%) | 1.000 |
|  | V106A/M | 17 (0.3%) | 2 (0.28%, 0.08–1.02%) | 15 (0.26%, 0.16–0.43%) | 1.000 |
|  | G190A/E/S | 13 (0.2%) | 4 (0.56%, 0.22–1.44%) | 9 (0.16%, 0.08–0.30%) | 0.510 |
|  | P225H | 11 (0.2%) | 3 (0.42%, 0.14–1.23%) | 8 (0.14%, 0.07–0.27%) | 0.617 |
|  | Y188C/H/L | 11 (0.2%) | 1 (0.14%, 0.02–0.79%) | 10 (0.17%, 0.09–0.32%) | 1.000 |
|  | K101E/P | 10 (0.2%) | 2 (0.28%, 0.08–1.02%) | 8 (0.14%, 0.07–0.27%) | 1.000 |
|  | L100I | 6 (0.1%) | 0 (0.00%, 0.00–0.54%) | 6 (0.10%, 0.05–0.23%) | 1.000 |
| **NRTI** | M184I/IV/V | 33 (0.5%) | 4 (0.56%, 0.22–1.44%) | 29 (0.50%, 0.35–0.72%) | 1.000 |
|  | K219E/N/Q/R | 18 (0.3%) | 1 (0.14%, 0.02–0.79%) | 17 (0.30%, 0.18–0.47%) | 1.000 |
|  | T215D/F/I/S/SY/Y | 14 (0.2%) | 0 (0.00%, 0.00–0.54%) | 14 (0.24%, 0.15–0.41%) | 1.000 |
|  | M41L | 10 (0.2%) | 1 (0.14%, 0.02–0.79%) | 9 (0.16%, 0.08–0.30%) | 1.000 |
|  | D67E/G/N | 9 (0.1%) | 1 (0.14%, 0.02–0.79%) | 8 (0.14%, 0.07–0.27%) | 1.000 |
|  | K70E/R | 9 (0.1%) | 0 (0.00%, 0.00–0.54%) | 9 (0.16%, 0.08–0.30%) | 1.000 |
|  | K65R | 9 (0.1%) | 0 (0.00%, 0.00–0.54%) | 9 (0.16%, 0.08–0.30%) | 1.000 |
|  | F77L | 8 (0.1%) | 0 (0.00%, 0.00–0.54%) | 8 (0.14%, 0.07–0.27%) | 1.000 |
|  | L210W | 8 (0.1%) | 1 (0.14%, 0.02–0.79%) | 7 (0.12%, 0.06–0.25%) | 1.000 |
|  | Y115F | 5 (0.1%) | 0 (0.00%, 0.00–0.54%) | 5 (0.09%, 0.04–0.20%) | 1.000 |
|  | V75A/AT/M | 4 (0.1%) | 1 (0.14%, 0.02–0.79%) | 3 (0.05%, 0.02–0.15%) | 1.000 |
| **PI** | M46I/L | 31 (0.5%) | 7 (0.98%, 0.48–2.02%) | 24 (0.42%, 0.28–0.62%) | 0.545 |
|  | I85V | 5 (0.1%) | 1 (0.14%, 0.02–0.79%) | 4 (0.07%, 0.03–0.18%) | 1.000 |
|  | F53L | 4 (0.1%) | 0 (0.00%, 0.00–0.54%) | 4 (0.07%, 0.03–0.18%) | 1.000 |

**Supplementary Table 4F. Prevalence of transmitted drug resistance (TDR) overall and by drug class, stratified by population group (corresponds to Figure 2A).**

| **Population group** | **N** | **Overall TDR,**  **n (%, 95% CI)** | **NNRTI,**  **n (%, 95% CI)** | **NRTI,**  **n (%, 95% CI)** | **PI,**  **n (%, 95% CI)** |
| --- | --- | --- | --- | --- | --- |
| **Resident migrants** | 2811 | 159 (5.7%, 4.9–6.6%) | 101 (3.6%, 3.0–4.3%) | 46 (1.6%, 1.2–2.2%) | 28 (1.0%, 0.7–1.4%) |
| **Shanghai residents** | 2319 | 98 (4.2%, 3.5–5.1%) | 44 (1.9%, 1.4–2.5%) | 34 (1.5%, 1.1–2.0%) | 24 (1.0%, 0.7–1.5%) |
| **Temporary migrants** | 2408 | 126 (5.2%, 4.4–6.2%) | 70 (2.9%, 2.3–3.7%) | 47 (2.0%, 1.5–2.6%) | 23 (1.0%, 0.6–1.4%) |

**Supplementary Table 4G. Annual prevalence of transmitted drug resistance (TDR) by drug class within each population group, 2018–2024 (corresponds to Figure 2B).**

| ***Resident migrants*** | | | | |
| --- | --- | --- | --- | --- |
| **Year** | **N** | **NNRTI, n (%, 95% CI)** | **NRTI, n (%, 95% CI)** | **PI, n (%, 95% CI)** |
| **2018** | 243 | 5 (2.1%, 0.9–4.7%) | 8 (3.3%, 1.7–6.4%) | 2 (0.8%, 0.2–3.0%) |
| **2019** | 437 | 12 (2.7%, 1.6–4.7%) | 5 (1.1%, 0.5–2.7%) | 8 (1.8%, 0.9–3.6%) |
| **2020** | 308 | 7 (2.3%, 1.1–4.6%) | 3 (1.0%, 0.3–2.8%) | 3 (1.0%, 0.3–2.8%) |
| **2021** | 472 | 14 (3.0%, 1.8–4.9%) | 4 (0.8%, 0.3–2.2%) | 7 (1.5%, 0.7–3.0%) |
| **2022** | 379 | 12 (3.2%, 1.8–5.5%) | 4 (1.1%, 0.4–2.7%) | 2 (0.5%, 0.1–1.9%) |
| **2023** | 526 | 29 (5.5%, 3.9–7.8%) | 9 (1.7%, 0.9–3.2%) | 5 (1.0%, 0.4–2.2%) |
| **2024** | 446 | 22 (4.9%, 3.3–7.4%) | 13 (2.9%, 1.7–4.9%) | 1 (0.2%, 0.0–1.3%) |
| ***Shanghai residents*** | | | | |
| **Year** | **N** | **NNRTI, n (%, 95% CI)** | **NRTI, n (%, 95% CI)** | **PI, n (%, 95% CI)** |
| **2018** | 379 | 9 (2.4%, 1.3–4.5%) | 6 (1.6%, 0.7–3.4%) | 5 (1.3%, 0.6–3.1%) |
| **2019** | 404 | 4 (1.0%, 0.4–2.5%) | 5 (1.2%, 0.5–2.9%) | 1 (0.2%, 0.0–1.4%) |
| **2020** | 244 | 1 (0.4%, 0.1–2.3%) | 5 (2.0%, 0.9–4.7%) | 3 (1.2%, 0.4–3.6%) |
| **2021** | 350 | 6 (1.7%, 0.8–3.7%) | 4 (1.1%, 0.4–2.9%) | 5 (1.4%, 0.6–3.3%) |
| **2022** | 310 | 9 (2.9%, 1.5–5.4%) | 3 (1.0%, 0.3–2.8%) | 3 (1.0%, 0.3–2.8%) |
| **2023** | 382 | 6 (1.6%, 0.7–3.4%) | 8 (2.1%, 1.1–4.1%) | 5 (1.3%, 0.6–3.0%) |
| **2024** | 250 | 9 (3.6%, 1.9–6.7%) | 3 (1.2%, 0.4–3.5%) | 2 (0.8%, 0.2–2.9%) |
| ***Temporary migrants*** | | | | |
| **Year** | **N** | **NNRTI, n (%, 95% CI)** | **NRTI, n (%, 95% CI)** | **PI, n (%, 95% CI)** |
| **2018** | 483 | 21 (4.3%, 2.9–6.6%) | 14 (2.9%, 1.7–4.8%) | 7 (1.4%, 0.7–3.0%) |
| **2019** | 456 | 7 (1.5%, 0.7–3.1%) | 6 (1.3%, 0.6–2.8%) | 6 (1.3%, 0.6–2.8%) |
| **2020** | 278 | 4 (1.4%, 0.6–3.6%) | 5 (1.8%, 0.8–4.1%) | 1 (0.4%, 0.1–2.0%) |
| **2021** | 353 | 13 (3.7%, 2.2–6.2%) | 3 (0.8%, 0.3–2.5%) | 3 (0.8%, 0.3–2.5%) |
| **2022** | 269 | 8 (3.0%, 1.5–5.8%) | 1 (0.4%, 0.1–2.1%) | 3 (1.1%, 0.4–3.2%) |
| **2023** | 330 | 8 (2.4%, 1.2–4.7%) | 10 (3.0%, 1.7–5.5%) | 3 (0.9%, 0.3–2.6%) |
| **2024** | 239 | 9 (3.8%, 2.0–7.0%) | 8 (3.3%, 1.7–6.5%) | 0 (0.0%, 0.0–1.6%) |

**Supplementary Table 5. Summary of molecular transmission network metrics across four genetic distance (GD) thresholds for the overall dataset and major HIV-1 genotypes.**

| **Metric** | **GD = 0.005** | **GD = 0.010** | **GD = 0.012*** | **GD = 0.015** |
| --- | --- | --- | --- | --- |
| ***Overall*** | | | | |
| Total clustered individuals, n (%) | 1236 (16.4%) | 2612 (34.7%) | **3132 (41.5%)** | 3931 (52.1%) |
| Total number of transmission clusters | 418 | 660 | **695** | 677 |
| Maximum cluster size | 19 | 398 | **765** | 1429 |
| TDR-containing clusters (≥1 TDR case) | 34 | 66 | **72** | 76 |
| TDR transmission clusters (≥2 TDR cases) | 12 | 21 | **19** | 20 |
| Resident migrant clustering proportion, % (n/N) | 18.4% (516/2811) | 37.2% (1045/2811) | **44.1% (1239/2811)** | 54.5% (1532/2811) |
| High-degree nodes (degree ≥5) among clustered resident migrants, % (n/N) | 7.2% (37/516) | 19.7% (206/1045) | **25.3% (313/1239)** | 33.2% (508/1532) |
| TDR–TDR edges involving resident migrants, % (n/N) | 77.8% (14/18) | 82.1% (46/56) | **74.3% (55/74)** | 74.0% (71/96) |
| TDR transmission clusters involving TDR-positive resident migrants, % (n/N) | 66.7% (8/12) | 81.0% (17/21) | **78.9% (15/19)** | 80.0% (16/20) |
| ***CRF07_BC*** | | | | |
| Total clustered individuals, n (%) | 600 (18.9%) | 1282 (40.5%) | **1577 (49.8%)** | 2024 (63.9%) |
| Total number of transmission clusters | 189 | 255 | **237** | 183 |
| Maximum cluster size | 19 | 396 | **762** | 1420 |
| TDR-containing clusters (≥1 TDR case) | 16 | 26 | **25** | 20 |
| TDR transmission clusters (≥2 TDR cases) | 3 | 9 | **8** | 4 |
| Resident migrant clustering proportion, % (n/N) | 20.9% (256/1224) | 43.1% (527/1224) | **51.6% (632/1224)** | 63.9% (782/1224) |
| High-degree nodes (degree ≥5) among clustered resident migrants, % (n/N) | 10.9% (28/256) | 23.7% (125/527) | **30.5% (193/632)** | 41.3% (323/782) |
| TDR–TDR edges involving resident migrants, % (n/N) | 100.0% (5/5) | 87.5% (28/32) | **77.3% (34/44)** | 76.4% (42/55) |
| TDR transmission clusters involving TDR-positive resident migrants, % (n/N) | 100.0% (3/3) | 77.8% (7/9) | **75.0% (6/8)** | 75.0% (3/4) |
| ***CRF01_AE*** | | | | |
| Total clustered individuals, n (%) | 364 (13.7%) | 787 (29.5%) | **922 (34.6%)** | 1160 (43.5%) |
| Total number of transmission clusters | 134 | 247 | **281** | 311 |
| Maximum cluster size | 14 | 23 | **26** | 77 |
| TDR-containing clusters (≥1 TDR case) | 10 | 28 | **31** | 39 |
| TDR transmission clusters (≥2 TDR cases) | 6 | 8 | **7** | 11 |
| Resident migrant clustering proportion, % (n/N) | 15.0% (155/1031) | 31.9% (329/1031) | **37.3% (385/1031)** | 47.0% (485/1031) |
| High-degree nodes (degree ≥5) among clustered resident migrants, % (n/N) | 5.2% (8/155) | 16.4% (54/329) | **19.7% (76/385)** | 24.7% (120/485) |
| TDR–TDR edges involving resident migrants, % (n/N) | 75.0% (6/8) | 69.2% (9/13) | **63.2% (12/19)** | 66.7% (20/30) |
| TDR transmission clusters involving TDR-positive resident migrants, % (n/N) | 66.7% (4/6) | 87.5% (7/8) | **85.7% (6/7)** | 81.8% (9/11) |
| ***CRF55_01B*** | | | | |
| Total clustered individuals, n (%) | 61 (22.5%) | 122 (45.0%) | **143 (52.8%)** | 161 (59.4%) |
| Total number of transmission clusters | 22 | 34 | **40** | 40 |
| Maximum cluster size | 6 | 14 | **15** | 19 |
| TDR-containing clusters (≥1 TDR case) | 2 | 4 | **4** | 5 |
| TDR transmission clusters (≥2 TDR cases) | 1 | 1 | **1** | 1 |
| Resident migrant clustering proportion, % (n/N) | 29.0% (31/107) | 53.3% (57/107) | **62.6% (67/107)** | 71.0% (76/107) |
| High-degree nodes (degree ≥5) among clustered resident migrants, % (n/N) | 0.0% (0/31) | 14.0% (8/57) | **25.4% (17/67)** | 28.9% (22/76) |
| TDR–TDR edges involving resident migrants, % (n/N) | 100.0% (3/3) | 100.0% (6/6) | **100.0% (6/6)** | 100.0% (6/6) |
| TDR transmission clusters involving TDR-positive resident migrants, % (n/N) | 100.0% (1/1) | 100.0% (1/1) | **100.0% (1/1)** | 100.0% (1/1) |
| *TDR: transmitted drug resistance. *GD = 0.012 substitutions/site was the primary threshold used for the mixed-genotype network reported in the main text; the remaining thresholds were evaluated as sensitivity analyses. A visual summary of the resident migrant-related metrics is provided in Supplementary Figure 5.* | | | | |
